# 5-Iodo-2,7-dimethyl-3-phenyl­sulfinyl-1-benzofuran

**DOI:** 10.1107/S1600536808001797

**Published:** 2008-01-23

**Authors:** Hong Dae Choi, Pil Ja Seo, Byeng Wha Son, Uk Lee

**Affiliations:** a Department of Chemistry, Dongeui University, San 24 Kaya-dong, Busanjin-gu, Busan 614-714, Republic of Korea; b Department of Chemistry, Pukyong National University, 599-1 Daeyeon 3-dong, Nam-gu, Busan 608-737, Republic of Korea

## Abstract

The title compound, C_16_H_13_IO_2_S, was prepared by the oxidation of 5-iodo-2,7-dimethyl-3-phenyl­sulfanyl-1-benzofuran using 3-chloro­perbenzoic acid. The O atom and the phenyl group of the phenyl­sulfinyl substituent lie on opposite sides of the plane of the benzofuran system. The phenyl ring is nearly perpendicular to the plane of the benzofuran fragment [89.15 (5)°]. The crystal structure is stabilized by an I⋯O halogen bond [I⋯O = 3.177 (2) Å and C—I⋯O = 175.68 (6)°] linking mol­ecules into centrosymmetric dimers and by a weak C—H⋯π inter­action between a phenyl H atom and the furan ring of the benzofuran system.

## Related literature

For the crystal structures of similar 5-iodo-2-methyl-1-benzofuran compounds, see: Choi *et al.* (2007*a* ▶,*b* ▶). For a review of halogen bonding, see: Politzer *et al.* (2007 ▶).
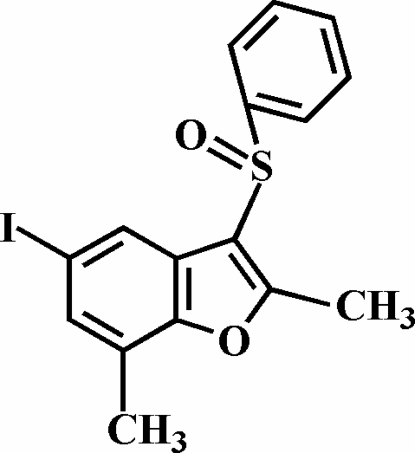


## Experimental

### 

#### Crystal data

C_16_H_13_IO_2_S
*M* *_r_* = 396.22
Monoclinic, 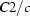

*a* = 24.4683 (8) Å
*b* = 8.1686 (3) Å
*c* = 16.2345 (5) Å
β = 113.015 (1)°
*V* = 2986.54 (17) Å^3^
*Z* = 8
Mo *K*α radiation
μ = 2.28 mm^−1^
*T* = 173 (2) K
0.40 × 0.40 × 0.20 mm

#### Data collection

Bruker SMART CCD diffractometer
Absorption correction: multi-scan (*SADABS*; Sheldrick, 2000 ▶) *T*_min_ = 0.412, *T*_max_ = 0.640
8756 measured reflections
3261 independent reflections
3063 reflections with *I* > 2σ(*I*)
*R*_int_ = 0.013

#### Refinement

*R*[*F* ^2^ > 2σ(*F* ^2^)] = 0.021
*wR*(*F* ^2^) = 0.053
*S* = 1.17
3261 reflections
183 parameters
H-atom parameters constrained
Δρ_max_ = 0.29 e Å^−3^
Δρ_min_ = −0.80 e Å^−3^

### 

Data collection: *SMART* (Bruker, 1997 ▶); cell refinement: *SAINT* (Bruker, 1997 ▶); data reduction: *SAINT*; program(s) used to solve structure: *SHELXS97* (Sheldrick, 2008 ▶); program(s) used to refine structure: *SHELXL97* (Sheldrick, 2008 ▶); molecular graphics: *ORTEP-3* (Farrugia, 1997 ▶) and *DIAMOND* (Brandenburg, 1998 ▶); software used to prepare material for publication: *SHELXL97*.

## Supplementary Material

Crystal structure: contains datablocks global, I. DOI: 10.1107/S1600536808001797/gk2130sup1.cif

Structure factors: contains datablocks I. DOI: 10.1107/S1600536808001797/gk2130Isup2.hkl

Additional supplementary materials:  crystallographic information; 3D view; checkCIF report

## Figures and Tables

**Table 1:** Hydrogen-bond geometry (Å, °) *Cg* is the centroid of the furan ring.

*D*—H⋯*A*	*D*—H	H⋯*A*	*D*⋯*A*	*D*—H⋯*A*
C13—H13⋯*Cg*^i^	0.95	2.82	3.576 (3)	137

Symmetry code: (i) 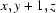.

**Fig. 1.**
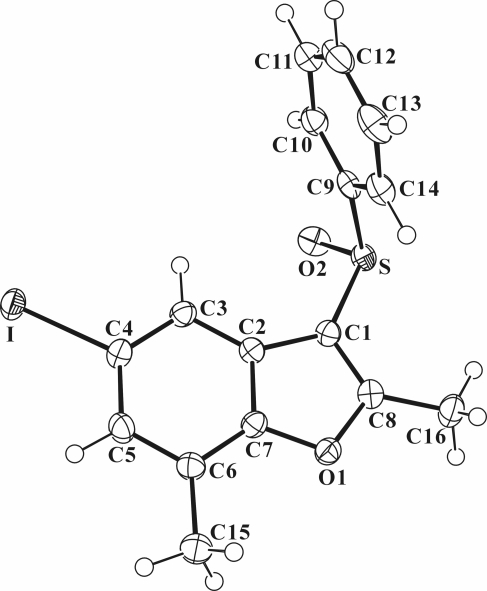
The molecular structure of the title compound, showing displacement ellipsoids drawn at the 50% probability level.

**Fig. 2.**
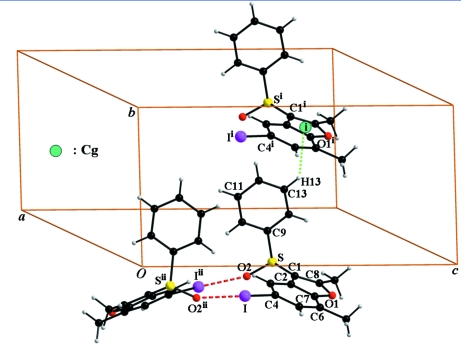
C—H···π interaction and I···O halogen bond (dotted lines) in crystals of the title compound. [Symmetry code: (i) x, y + 1, z; (ii) -x, y, -z + 1/2.]

## supplementary crystallographic information

### Comment

As part of our continuing studies on the synthesis and structure of 5-iodo-2-methyl-1-benzofuran derivatives, the crystal structures of 5-iodo-2-methyl-3-phenylsulfinyl-1-benzofuran (Choi *et al.*, 2007*a*) and 5-iodo-2-methyl-3-methylsulfinyl-1-benzofuran (Choi *et al.*, 2007*b*) have been described to the literatures. Herein we report the molecular and crystal structure of the title compound, 2,7-dimethyl-5-iodo-3-phenylsulfinyl-1-benzofuran (Fig. 1).

The benzofuran unit is essentially planar, with a mean deviation of 0.012 Å from the least-squares plane defined by the nine constituent atoms. The phenyl ring (C9—C14) is almost perpendicular to the plane of the benzofuran system [89.15 (5)°] and is tilted slightly towards it. The molecular packing (Fig. 2) is stabilized by a C—H···π interaction between the phenyl H atom and the furan ring of the benzofuran uint, with a C13—H13···*Cg*^i^ separation of 2.82 Å (Fig. 2 and Table 1; *Cg* is the centroid of C1/C2/C7/O1/C8 furan ring, symmetry code as in Fig. 2). The molecular packing (Fig. 2) is further stabilized by an I···O halogen bond (Politzer *et al.*, 2007) between the iodine atom and the oxygen of a neighbouring S?O unit, with an C—I···O2^ii^ distance of 3.177 (2) Å (symmetry code as in Fig. 2).

### Experimental

3-Chloroperbenzoic acid (77%, 123 mg, 0.55 mmol) was added in small portions to a stirred solution of 2,7-dimethyl-5-iodo-3-phenylsulfanyl-1-benzofuran (190 mg, 0.5 mmol) in dichloromethane (20 ml) at 273 K. After being stirred at room temperature for 2 h, the mixture was washed with saturated sodium bicarbonate solution and the organic layer was separated, dried over magnesium sulfate, filtered and concentrated in vacuum. The residue was purified by column chromatography (hexane-ethyl acetate, 2:1 *v*/*v*) to afford the title compound as a colorless solid [yield 80%, m.p. 450–451 K; *R*_f_ = 0.41 (hexane-ethyl acetate, 2:1 *v*/*v*)]. Single crystals suitable for X-ray diffraction were prepared by slow evaporation of a dilute solution of the title compound in benzene at room temperature.

### Refinement

All H atoms were positioned geometrically and refined using a riding model, with C—H = 0.95 Å for aromatic H atoms and 0.98 Å for methyl H atoms, respectively, and with *U*_iso_(H) = 1.2Ueq(C) for aromatic H atoms and *U*_iso_(H) = 1.5Ueq(C) for methyl H atoms.

### Crystal data

**Table:**

C_16_H_13_IO_2_S	*F*_000_ = 1552
*M**_r_* = 396.22	*D*_x_ = 1.762 Mg m^−^^3^
Monoclinic, *C*2/*c*	Mo *K*α radiation λ = 0.71073 Å
Hall symbol: -C 2yc	Cell parameters from 7108 reflections
*a* = 24.4683 (8) Å	θ = 2.6–28.3º
*b* = 8.1686 (3) Å	µ = 2.28 mm^−^^1^
*c* = 16.2345 (5) Å	*T* = 173 (2) K
β = 113.015 (1)º	Block, colorless
*V* = 2986.54 (17) Å^3^	0.40 × 0.40 × 0.20 mm
*Z* = 8	

### Data collection

**Table:**

Bruker SMART CCD diffractometer	3261 independent reflections
Radiation source: fine-focus sealed tube	3063 reflections with *I* > 2σ(*I*)
Monochromator: graphite	*R*_int_ = 0.013
Detector resolution: 10.0 pixels mm^-1^	θ_max_ = 27.0º
*T* = 173(2) K	θ_min_ = 2.7º
φ and ω scans	*h* = −30→31
Absorption correction: multi-scan(SADABS; Sheldrick, 2000)	*k* = −10→6
*T*_min_ = 0.412, *T*_max_ = 0.640	*l* = −20→20
8756 measured reflections	

### Refinement

**Table:**

Refinement on *F*^2^	Secondary atom site location: difference Fourier map
Least-squares matrix: full	Hydrogen site location: inferred from neighbouring sites
*R*[*F*^2^ > 2σ(*F*^2^)] = 0.021	H-atom parameters constrained
*wR*(*F*^2^) = 0.053	*w* = 1/[σ^2^(*F*_o_^2^) + (0.0277*P*)^2^ + 2.4985*P*] where *P* = (*F*_o_^2^ + 2*F*_c_^2^)/3
*S* = 1.17	(Δ/σ)_max_ < 0.001
3261 reflections	Δρ_max_ = 0.29 e Å^−^^3^
183 parameters	Δρ_min_ = −0.80 e Å^−^^3^
Primary atom site location: structure-invariant direct methods	Extinction correction: none

### Special details

**Table:**

Geometry. All e.s.d.'s (except the e.s.d. in the dihedral angle between two l.s. planes) are estimated using the full covariance matrix. The cell e.s.d.'s are taken into account individually in the estimation of e.s.d.'s in distances, angles and torsion angles; correlations between e.s.d.'s in cell parameters are only used when they are defined by crystal symmetry. An approximate (isotropic) treatment of cell e.s.d.'s is used for estimating e.s.d.'s involving l.s. planes.
Refinement. Refinement of *F*^2^ against ALL reflections. The weighted *R*-factor *wR* and goodness of fit *S* are based on *F*^2^, conventional *R*-factors *R* are based on *F*, with *F* set to zero for negative *F*^2^. The threshold expression of *F*^2^ > σ(*F*^2^) is used only for calculating *R*-factors(gt) *etc*. and is not relevant to the choice of reflections for refinement. *R*-factors based on *F*^2^ are statistically about twice as large as those based on *F*, and *R*- factors based on ALL data will be even larger.

### Fractional atomic coordinates and isotropic or equivalent isotropic displacement parameters (Å^2^)

**Table:**

	*x*	*y*	*z*	*U*_iso_*/*U*_eq_	
I	−0.081673 (5)	−0.158666 (17)	0.292529 (8)	0.02880 (6)	
S	0.20287 (2)	−0.04894 (6)	0.48963 (3)	0.02319 (10)	
O1	0.13474 (6)	−0.23728 (17)	0.65079 (9)	0.0242 (3)	
O2	0.18514 (7)	−0.13130 (18)	0.40076 (10)	0.0307 (3)	
C1	0.15689 (8)	−0.1211 (2)	0.54224 (13)	0.0217 (4)	
C2	0.09364 (8)	−0.1507 (2)	0.50573 (13)	0.0208 (4)	
C3	0.04657 (8)	−0.1279 (2)	0.42283 (13)	0.0232 (4)	
H3	0.0524	−0.0787	0.3738	0.028*	
C4	−0.00869 (9)	−0.1805 (2)	0.41583 (13)	0.0246 (4)	
C5	−0.01857 (9)	−0.2510 (3)	0.48745 (13)	0.0279 (4)	
H5	−0.0576	−0.2842	0.4791	0.034*	
C6	0.02741 (9)	−0.2734 (3)	0.57055 (13)	0.0269 (4)	
C7	0.08257 (8)	−0.2231 (2)	0.57559 (12)	0.0220 (4)	
C8	0.17897 (9)	−0.1754 (2)	0.62816 (13)	0.0235 (4)	
C9	0.17569 (8)	0.1567 (2)	0.46631 (14)	0.0227 (4)	
C10	0.16105 (9)	0.2173 (3)	0.38100 (14)	0.0277 (4)	
H10	0.1614	0.1477	0.3343	0.033*	
C11	0.14575 (10)	0.3820 (3)	0.36411 (16)	0.0340 (5)	
H11	0.1354	0.4249	0.3055	0.041*	
C12	0.14560 (9)	0.4826 (3)	0.43195 (17)	0.0357 (5)	
H12	0.1352	0.5947	0.4201	0.043*	
C13	0.16055 (10)	0.4211 (3)	0.51723 (17)	0.0357 (5)	
H13	0.1601	0.4913	0.5636	0.043*	
C14	0.17623 (9)	0.2583 (3)	0.53581 (14)	0.0281 (4)	
H14	0.1871	0.2165	0.5947	0.034*	
C15	0.01764 (11)	−0.3447 (3)	0.64905 (16)	0.0429 (6)	
H15A	0.0377	−0.4508	0.6647	0.064*	
H15B	−0.0250	−0.3595	0.6333	0.064*	
H15C	0.0338	−0.2702	0.7003	0.064*	
C16	0.23960 (9)	−0.1798 (3)	0.69950 (14)	0.0317 (5)	
H16A	0.2685	−0.1459	0.6747	0.048*	
H16B	0.2487	−0.2914	0.7231	0.048*	
H16C	0.2417	−0.1052	0.7479	0.048*	

### Atomic displacement parameters (Å^2^)

**Table:**

	*U*^11^	*U*^22^	*U*^33^	*U*^12^	*U*^13^	*U*^23^
I	0.02196 (8)	0.03709 (9)	0.02313 (8)	0.00094 (5)	0.00425 (6)	0.00074 (5)
S	0.0188 (2)	0.0262 (2)	0.0266 (2)	0.00015 (17)	0.01100 (18)	−0.00063 (18)
O1	0.0225 (6)	0.0283 (7)	0.0211 (6)	0.0003 (5)	0.0079 (5)	0.0015 (5)
O2	0.0394 (8)	0.0282 (7)	0.0312 (8)	−0.0009 (6)	0.0210 (7)	−0.0059 (6)
C1	0.0205 (9)	0.0227 (9)	0.0224 (9)	0.0012 (7)	0.0090 (7)	−0.0004 (7)
C2	0.0201 (9)	0.0212 (9)	0.0225 (9)	0.0011 (7)	0.0100 (7)	−0.0011 (7)
C3	0.0233 (9)	0.0262 (10)	0.0207 (9)	0.0017 (7)	0.0092 (7)	0.0022 (7)
C4	0.0217 (9)	0.0288 (10)	0.0212 (9)	0.0021 (7)	0.0060 (7)	−0.0005 (7)
C5	0.0205 (9)	0.0375 (11)	0.0267 (10)	−0.0033 (8)	0.0102 (8)	−0.0005 (8)
C6	0.0260 (10)	0.0324 (10)	0.0241 (9)	−0.0027 (8)	0.0118 (8)	0.0008 (8)
C7	0.0225 (9)	0.0240 (9)	0.0194 (8)	0.0020 (7)	0.0082 (7)	−0.0002 (7)
C8	0.0219 (9)	0.0238 (9)	0.0246 (9)	0.0008 (7)	0.0090 (8)	−0.0033 (7)
C9	0.0161 (8)	0.0234 (9)	0.0295 (10)	−0.0043 (7)	0.0101 (7)	−0.0038 (7)
C10	0.0275 (10)	0.0283 (10)	0.0266 (10)	−0.0060 (8)	0.0099 (8)	−0.0043 (8)
C11	0.0279 (10)	0.0316 (11)	0.0369 (12)	−0.0067 (9)	0.0066 (9)	0.0046 (9)
C12	0.0272 (10)	0.0233 (10)	0.0561 (14)	−0.0050 (8)	0.0156 (10)	−0.0013 (9)
C13	0.0333 (11)	0.0303 (11)	0.0497 (13)	−0.0085 (9)	0.0230 (10)	−0.0144 (10)
C14	0.0274 (10)	0.0305 (11)	0.0290 (10)	−0.0073 (8)	0.0138 (8)	−0.0077 (8)
C15	0.0319 (12)	0.0681 (18)	0.0300 (12)	−0.0089 (11)	0.0136 (10)	0.0117 (11)
C16	0.0237 (10)	0.0408 (12)	0.0258 (10)	0.0015 (8)	0.0044 (8)	0.0007 (8)

### Geometric parameters (Å, °)

**Table:**

I—C4	2.105 (2)	C8—C16	1.483 (3)
I—O2^i^	3.177 (2)	C9—C10	1.380 (3)
S—O2	1.494 (2)	C9—C14	1.397 (3)
S—C1	1.759 (2)	C10—C11	1.394 (3)
S—C9	1.791 (2)	C10—H10	0.9500
O1—C8	1.369 (2)	C11—C12	1.375 (3)
O1—C7	1.384 (2)	C11—H11	0.9500
C1—C8	1.358 (3)	C12—C13	1.381 (3)
C1—C2	1.445 (3)	C12—H12	0.9500
C2—C7	1.396 (3)	C13—C14	1.384 (3)
C2—C3	1.400 (3)	C13—H13	0.9500
C3—C4	1.381 (3)	C14—H14	0.9500
C3—H3	0.9500	C15—H15A	0.9800
C4—C5	1.400 (3)	C15—H15B	0.9800
C5—C6	1.390 (3)	C15—H15C	0.9800
C5—H5	0.9500	C16—H16A	0.9800
C6—C7	1.382 (3)	C16—H16B	0.9800
C6—C15	1.503 (3)	C16—H16C	0.9800
			
C4—I—O2^i^	175.68 (6)	C6—C7—C2	125.0 (2)
O2—S—C1	108.58 (9)	O1—C7—C2	110.3 (2)
O2—S—C9	105.92 (9)	C1—C8—O1	111.0 (2)
C1—S—C9	99.30 (9)	C1—C8—C16	133.2 (2)
C8—O1—C7	106.6 (1)	O1—C8—C16	115.8 (2)
C8—C1—C2	107.4 (2)	C10—C9—C14	120.9 (2)
C8—C1—S	122.3 (2)	C10—C9—S	118.6 (2)
C2—C1—S	130.0 (2)	C14—C9—S	120.1 (2)
C7—C2—C3	119.3 (2)	C9—C10—C11	119.2 (2)
C7—C2—C1	104.7 (2)	C12—C11—C10	120.2 (2)
C3—C2—C1	136.0 (2)	C11—C12—C13	120.2 (2)
C4—C3—C2	116.7 (2)	C12—C13—C14	120.7 (2)
C3—C4—C5	122.8 (2)	C13—C14—C9	118.7 (2)
C3—C4—I	119.4 (1)	H15A—C15—H15B	109.5
C5—C4—I	117.8 (1)	H15A—C15—H15C	109.5
C6—C5—C4	121.5 (2)	H15B—C15—H15C	109.5
C7—C6—C5	114.8 (2)	H16A—C16—H16B	109.5
C7—C6—C15	122.7 (2)	H16A—C16—H16C	109.5
C5—C6—C15	122.5 (2)	H16B—C16—H16C	109.5
C6—C7—O1	124.7 (2)		
			
O2—S—C1—C8	130.7 (2)	C3—C2—C7—C6	−1.2 (3)
C9—S—C1—C8	−118.9 (2)	C1—C2—C7—C6	−179.7 (2)
O2—S—C1—C2	−42.4 (2)	C3—C2—C7—O1	178.9 (2)
C9—S—C1—C2	68.0 (2)	C1—C2—C7—O1	0.4 (2)
C8—C1—C2—C7	0.0 (2)	C2—C1—C8—O1	−0.5 (2)
S—C1—C2—C7	174.0 (2)	S—C1—C8—O1	−174.9 (1)
C8—C1—C2—C3	−178.1 (2)	C2—C1—C8—C16	179.7 (2)
S—C1—C2—C3	−4.1 (3)	S—C1—C8—C16	5.2 (3)
C7—C2—C3—C4	−0.3 (3)	C7—O1—C8—C1	0.7 (2)
C1—C2—C3—C4	177.6 (2)	C7—O1—C8—C16	−179.5 (2)
C2—C3—C4—C5	1.1 (3)	O2—S—C9—C10	−18.3 (2)
C2—C3—C4—I	−178.0 (1)	C1—S—C9—C10	−130.8 (2)
C3—C4—C5—C6	−0.5 (3)	O2—S—C9—C14	169.2 (2)
I—C4—C5—C6	178.5 (2)	C1—S—C9—C14	56.8 (2)
C4—C5—C6—C7	−0.8 (3)	C14—C9—C10—C11	−1.0 (3)
C4—C5—C6—C15	178.4 (2)	S—C9—C10—C11	−173.4 (2)
C5—C6—C7—O1	−178.4 (2)	C9—C10—C11—C12	0.3 (3)
C15—C6—C7—O1	2.4 (3)	C10—C11—C12—C13	0.0 (3)
C5—C6—C7—C2	1.7 (3)	C11—C12—C13—C14	0.4 (3)
C15—C6—C7—C2	−177.5 (2)	C12—C13—C14—C9	−1.1 (3)
C8—O1—C7—C6	179.40 (19)	C10—C9—C14—C13	1.3 (3)
C8—O1—C7—C2	−0.6 (2)	S—C9—C14—C13	173.6 (2)

Symmetry codes: (i) −*x*, *y*, −*z*+1/2.

### Hydrogen-bond geometry (Å, °)

**Table:**

*D*—H···*A*	*D*—H	H···*A*	*D*···*A*	*D*—H···*A*
C13—H13···Cg^ii^	0.95	2.82	3.576 (3)	137

Symmetry codes: (ii) *x*, *y*+1, *z*.
